# Inactivated *Akkermansia muciniphila* AKK PROBIO Preserves Intestinal Homeostasis and Ameliorates DSS-Induced Colitis in Mice

**DOI:** 10.3390/foods14234063

**Published:** 2025-11-27

**Authors:** Hongyan Zhang, Chunwen Liu, Yutian Huang, Xin Ma, Dayong Ren

**Affiliations:** 1 College of Food Science, Northeast Agricultural University, Harbin 150038, China; zhanghongyan_1016@163.com (H.Z.); 18281215992@163.com (C.L.); 2 College of Food Science and Engineering, Jilin Agricultural University, Changchun 130118, China; 15567064473@163.com; 3 State Key Laboratory of Bioreactor Engineering, East China University of Science and Technology, Shanghai 200237, China; pkartest@yeah.net

**Keywords:** inactivated *Akkermansia muciniphila* AKK PROBIO, ulcerative colitis, intestinal barrier, gut microbiota, short chain fatty acid

## Abstract

Ulcerative colitis (UC) is a chronic relapsing inflammatory bowel disease with escalating global incidence. Conventional therapies face limitations including substantial costs and adverse effects, while live probiotics pose safety risks in vulnerable populations. Postbiotics—inactivated microorganisms conferring health benefits—offer therapeutic potential without viable bacterial risks. This study investigated inactivated *Akkermansia muciniphila* AKK PROBIO in dextran sulfate sodium (DSS)-induced colitis mice. Inactivated AKK PROBIO significantly ameliorated disease manifestations, restoring body weight and food intake during days 10–14 (*p* < 0.01) and reducing Disease Activity Index scores (*p* < 0.0001). Treatment preserved colonic architecture, enhanced tight junction proteins (Claudin-1, Occludin, ZO-1), and elevated mucin 2 expression. Mechanistically, AKK PROBIO modulated inflammatory responses by increasing anti-inflammatory interleukin-10 (*p* < 0.05) while decreasing pro-inflammatory cytokines IL-1β, IL-6, and TNF-α (all *p* < 0.05). 16S rRNA sequencing revealed selective microbiota remodeling with enriched beneficial genera (*Ligilactobacillus*, *Lachnospiraceae_NK4A136_group*, *Bacteroides*, *Akkermansia*) and depleted pathobionts (*Escherichia-Shigella*). Functional profiling demonstrated enhanced microbial metabolic capacity in carbohydrate and amino acid metabolism pathways. Gas chromatography–mass spectrometry analysis confirmed elevated short-chain fatty acid production, particularly butyrate and isocaproate (*p* < 0.05). Correlation analyses revealed interconnected relationships among beneficial microbiota, short-chain fatty acids, and anti-inflammatory mediators, while showing inverse associations with pro-inflammatory cytokines. In summary, our findings demonstrate that inactivated AKK PROBIO alleviates colitis, supporting its development as a safe, food-derived postbiotic.

## 1. Introduction

The gut serves as the body’s largest immune organ, accounting for approximately 70% of immune function. Due to its constant direct exposure to intestinal contents, the gut is prone to excessive immune responses to antigens, leading to abnormal accumulation and infiltration of immune cells, excessive secretion of pro-inflammatory cytokines, and overproduction of reactive oxygen species (ROS) [1]. If these pathological and physiological processes persist, they may ultimately result in tissue damage, ulcer formation, and even trigger ulcerative colitis (UC). UC is a chronic relapsing inflammatory bowel disease characterized by continuous mucosal inflammation extending proximally from the rectum, potentially involving partial or entire colonic segments. The clinical manifestations predominantly include abdominal pain, diarrhea, and rectal bleeding. Although its precise pathogenesis remains incompletely understood, current research consensus indicates that multifactorial interactions involving environmental triggers, genetic predisposition, microbial dysbiosis, and immune dysregulation collectively contribute to the initiation and progression of UC [2]. Recent epidemiological studies have revealed a persistent upward trajectory in the global incidence of UC, with particularly striking annual growth rates of 14.9% in Brazil and 4.8% in China’s Taiwan region. Clinical treatment mainly depends on drugs such as aminosalicylic acid, glucocorticoid and biological agents, and severe patients need surgical intervention [3]. However, the chronic relapsing course of UC necessitates long-term treatment, wherein conventional therapies pose significant challenges due to their substantial financial burden and considerable adverse effects. Consequently, it is of great significance to develop safe, effective and economical alternative therapy.

The intestinal microbiota plays a critical role in the pathogenesis and recurrence of UC. Dysbiosis disrupts host–microbial interactions, leading to impaired colonic epithelial barrier function. This disruption triggers and perpetuates inflammation by dysregulating cytokine activity, thereby increasing disease susceptibility. A marked reduction in gut microbiota diversity is a hallmark of UC [4]. Probiotics, as live microbial preparations, have emerged as a significant adjunctive therapeutic option for gastrointestinal disorders by conferring host health benefits through multiple mechanisms when administered in adequate amounts, including enhancement of intestinal epithelial barrier function, immunomodulation, and restoration of microbial homeostasis [5]. Compared with healthy individuals, Earley et al. (2019) quantitatively analyzed the abundance of *Akkermansia* the colonic mucus of UC patients and found it was significantly reduced in four regions: the cecum, transverse colon, left colon, and rectum [6]. Furthermore, *Lactobacillus paracasei* BNCC345679 alleviates DSS-induced colitis-related damage to the gut barrier by increasing the abundance of *Akkermansia and Lactobacillus* [7]. It is noteworthy, however, that the therapeutic efficacy of probiotics is intrinsically linked to their acid and bile salt tolerance as well as intestinal colonization efficiency [8]. Clinical studies have revealed that probiotics may pose potential safety risks in specific populations, particularly critically ill patients. For instance, a randomized double-blind controlled trial involving 298 patients with severe acute pancreatitis demonstrated a significantly higher mortality rate in the probiotic group (16%) compared to the placebo group (6%) [9]. These safety concerns have restricted the clinical application of live bacterial preparations in certain high-risk populations.

Against this backdrop, postbiotics have emerged as a novel therapeutic strategy garnering widespread attention. According to the 2021 consensus definition by the International Scientific Association for Probiotics and Prebiotics (ISAPP), postbiotics refer to “preparations of inactivated microorganisms and/or their components that confer documented health benefits” [10]. Compared to probiotics and prebiotics, postbiotics contain no live microorganisms. This not only avoids the potential safety risks associated with live bacteria but also offers advantages such as a longer shelf life, easier production and storage, and the ability to pass through stomach acid effectively to function in the gut. Numerous animal studies have demonstrated the superior efficacy of postbiotics in modulating gut microbiota. For instance, inactivated preparations of *Bifidobacterium adolescentis* B8598 exhibited significantly greater regulatory effects on fecal microbial β-diversity, community structure, and metagenomic functionality compared to their live counterparts when treating UC mouse models [2]. Similarly, inactivated *Saccharomyces boulardii* has demonstrated enhanced anti-inflammatory and microbiota-modulating capacities compared to its viable form [11]. These findings suggest that postbiotics may more effectively activate host immunomodulatory pathways and microbial homeostasis restoration mechanisms through their specific components, such as cell wall polysaccharides and metabolic byproducts [12]. Notably, the absence of live bacterial colonization risk makes postbiotics particularly suitable for immunocompromised patients and critical care populations due to their enhanced safety profile. Current evidence suggests that postbiotic administration may represent a potential therapeutic strategy for restoring intestinal homeostasis in colitis patients [13].

*Akkermansia muciniphila* AKK PROBIO (AKK PROBIO) is a probiotic strain isolated from the intestinal tract of healthy adults, and its whole-genome sequencing and toxicological evaluations have confirmed its safety [14]. Previous research findings have demonstrated that this strain exhibits preventive efficacy against acute gout and alleviates colorectal cancer in mouse models; additionally, weight loss clinical trials not only verified its significant weight reduction effect but also unexpectedly observed that AKK PROBIO could improve anxiety, depression, and sleep disturbances via the gut–brain axis pathway [15,16,17]. Notably, current research on AKK PROBIO primarily focuses on live bacterial preparations, while explorations into the biological functions of its inactivated formulations remain relatively limited.

This study represents the first systematic evaluation of the ameliorative effects of inactivated AKK PROBIO formulation on UC. Numerous clinical studies have demonstrated that the live form of *A. muciniphila* (AKK) exerts multiple probiotic benefits, including alleviation of intestinal inflammatory responses [18], improvement of age-related cognitive dysfunction [19], regulation of lipid metabolism [20], and maintenance of immune homeostasis [21]. Notably, a groundbreaking study revealed that pasteurized, inactivated AKK strains exhibited superior efficacy compared to their live counterparts in certain metabolic disease models—specifically, in high-fat-diet-induced mouse models, the inactivated AKK group showed more pronounced suppression of weight gain and reduced fat accumulation, along with improved blood lipid profiles and significantly lower levels of blood glucose and insulin [22]. Therefore, we hypothesized that inactivated AKK PROBIO alleviates DSS-induced colitis by restoring intestinal barrier integrity and modulating intestinal homeostasis. Building on this foundation, the present research employs a multidimensional evaluation framework—encompassing histopathological analysis of intestinal tissues, inflammatory cytokine profiling, microbial community structure, and metabolomic characterization—to comprehensively investigate the therapeutic potential of inactivated AKK PROBIO in a DSS-induced murine model of UC. This research not only fills the gap in functional studies of AKK postbiotics in UC treatment but also provides critical theoretical support for the application of postbiotics as safe and stable novel microbial intervention agents in dietary supplementation and adjuvant therapy for UC.

## 2. Materials and Methods

### 2.1. Culture of Akkermansia muciniphila and Postbiotic Production

The AKK PROBIO strain was isolated from fecal samples of healthy adults. Its cultivation was performed using brain heart infusion (BHI) broth supplemented with 0.05% mucin type II under specific anaerobic conditions (37 °C, gas mixture of 10% H_2_, 10% CO_2_, and 80% N_2_). After 3 days of cultivation, its growth status was analyzed and characterized. Subsequently, the bacterial suspension was resuspended in PBS buffer and diluted to a concentration of 1 × 10^10^ CFU/mL; this suspension was then subjected to sterilization at 90 °C for 30 min to prepare postbiotics. To verify the inactivation effect of the probiotic, the inactivated bacterial suspension was inoculated into brain heart infusion (BHI) broth supplemented with 0.05% mucin type II and subjected to anaerobic cultivation at 37 °C for 3 days, which confirmed the absence of any colony formation.

### 2.2. Design of Animal Experiments

Twenty-four male C57BL/6J SPF-grade mice were purchased from Liaoning (Benxi, China) Changsheng Biotechnology Co., Ltd. (experimental animal production license number: SCXK (Liao) 2020-0001). The experimental protocol was approved after review by the Experimental Animal Ethics Committee of Jilin Agricultural University. Mice were subjected to 7 days of acclimation in a standard animal room (temperature: 25 ± 2 °C, humidity: 50 ± 5%, 12 h light/dark cycle). After acclimation, mice were evenly divided into 3 groups (8 mice per group) using the random number table method: Control group, DSS model group (DSS), and AKK PROBIO intervention group (AKK_PRO). The experimental period lasted 14 days. From Days 1 to 7, mice in the DSS and AKK_PRO groups (excluding the Control group) freely drank deionized water containing 2.5% DSS to induce an ulcerative colitis model. Throughout the entire experimental period, mice in the Control and DSS groups were orally gavaged with equal volumes of normal saline daily, while the AKK_PRO group received an inactivated probiotic suspension (dosage: 10 mL/kg·day). During the experiment, mouse body weight and food intake were recorded daily, and fecal characteristics (e.g., loose stool, mucous stool) and occult blood positivity were observed. Fresh feces from each group were collected 24 h before the end of the experiment. At the end of the experiment, mice were euthanized, and liver, spleen, colon tissues, and cecal contents were collected for subsequent experimental analysis.

### 2.3. Assessment of Disease Activity Index

Daily during the experimental period, food intake and body weight changes were recorded systematically, while fecal characteristics (looseness, adhesiveness) and fecal occult blood positivity were concurrently assessed. Using the predefined DAI scoring criteria, these parameters were quantitatively evaluated to calculate the Disease Activity Index (DAI) score. Table 1 delineates the grading criteria used for the DAI scores.

### 2.4. Histopathological Analysis

A 1 cm colon tissue sample was fixed with 4% paraformaldehyde, embedded in paraffin, and sectioned into 5 μm slices for H&E staining, followed by morphological observation under a light microscope.

For immunofluorescence staining, paraffin-embedded colon tissues were sectioned into 5 μm slices, followed by deparaffinization and washing steps. Sections were immersed in a citrate antigen retrieval buffer (0.1 M, pH 6.0), heated to boiling in a microwave oven on high power, then switched to low-power simmering mode and maintained at a gentle boil for 10–15 min for antigen retrieval. After retrieval, the sections were allowed to cool naturally to room temperature in the retrieval solution. The sections were blocked with 10% goat serum for 30 min and then incubated with primary antibodies at 37 °C for 2 h. After PBS washing, the sections were incubated with HRP-conjugated polyclonal goat anti-rabbit IgG secondary antibodies (1:200 dilution) at 37 °C for 1 h. DAPI was used for nuclear counterstaining, and images were acquired and analyzed using KF-Viewer software (v1.7.0.27).

### 2.5. Measurement of Cytokine Levels in Colon Tissue

A 0.1 g colon tissue sample was homogenized, subsequently centrifuged at 10,000× *g* for 20 min at 4 °C, and the supernatant was collected for subsequent use. The levels of IL-1β, IL-10, IL-6, and TNF-α in the supernatant were quantitatively detected using an ELISA kit (provided by Shanghai Enzyme-Linked Biotechnology Co., Ltd., Shanghai, China). The specific operational steps are as follows: Prior to the experiment, equilibrate the kit at room temperature for 20–30 min. Add standards, test samples, and blank controls to the corresponding wells, with three replicate wells for each. Add horseradish peroxidase-labeled detection antibody and incubate at 37 °C for 1 h. After washing 5 times, add substrates A and B, and incubate at 37 °C protected from light for 15 min. Add stop solution and measure the OD value at 450 nm.

### 2.6. Compositional Analysis of Gut Microbiota

Total genomic DNA from microbial communities was extracted following the manufacturer’s protocol of the E.Z.N.A.^®^ Soil DNA Kit (Omega Bio-tek, Norcross, GA, USA). The extracted DNA was used as a template for PCR amplification of the V3-V4 hypervariable regions of the 16S rRNA gene using barcoded primers 338F (5′-ACTCCTACGGGAGGCAGCAG-3′, with Illumina sequencing adapter and barcode sequences) and 806R (5′-GGACTACHVGGGTWTCTAAT-3′, with complementary adapter sequences). PCR products were separated by 2% agarose gel electrophoresis and purified using a DNA Gel Extraction Kit (PCR Clean-Up Kit, YuHua, China). Sequencing was performed on the Illumina NextSeq 2000 platform (Shanghai Majorbio Bio-pharm Technology Co., Ltd. (Shanghai, China)).

### 2.7. Quantification of Short-Chain Fatty Acids

The experiment employed cecal contents (50–100 mg) as samples. Following mechanical lysis (vortexing with steel balls or glass beads for 15 min), centrifugation (12,000× *g*, 4 °C, 10 min) was performed to collect the supernatant. To enhance metabolite stability and recovery, acidification with 15% phosphoric acid and liquid–liquid extraction with diethyl ether (supplemented with the internal standard 4-methylvaleric acid) were conducted. Gas chromatography–mass spectrometry (GC-MS) analysis was carried out using a Trace 1310 gas chromatograph (Thermo Fisher Scientific, Woonsocket, RI, USA) with helium as the carrier gas (flow rate: 1.2 mL/min), a split ratio of 10:1, and an injection volume of 1 μL (injector temperature: 250 °C). The column temperature program started at 90 °C, with gradual increases to 120 °C (10 °C/min), 150 °C (5 °C/min), and finally 250 °C (25 °C/min), totaling 2 min. Mass spectrometric detection was performed using an ISQ LT mass spectrometer (Thermo Fisher Scientific, USA) equipped with an electron impact (EI) ionization source. Metabolites were identified via NIST database matching and quantified using the internal standard method, targeting short-chain fatty acids such as acetic acid and propionic acid.

### 2.8. Statistical Analysis

All statistical analyses were performed using GraphPad Prism 9.5.1 software (GraphPad Software, Inc., La Jolla, CA, USA). Experimental design included 3–8 biological replicates per group, and data are presented as mean ± standard deviation (SD). Group difference analysis was conducted using one-way ANOVA, Kruskal–Wallis H test, Mann–Whitney U test, or Wilcoxon signed-rank test based on data distribution characteristics; correlation between variables was evaluated using the Spearman rank correlation coefficient. The statistical significance threshold was set at *p* < 0.05.

## 3. Results

### 3.1. Ingestion of Inactivated AKK PROBIO Alleviates Physiological Damage in DSS-Induced Mouse UC

**Figure 1:**
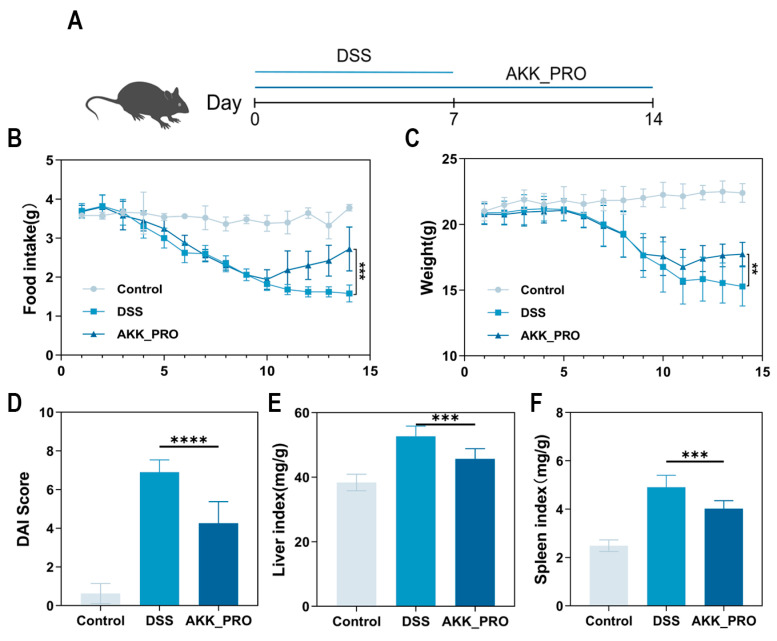
Inactivated AKK PROBIO ameliorates basic features of DSS-induced ulcerative colitis in mice. (**A**) Schematic illustration of the experimental design (*n* = 8 per group). (**B**) Daily food intake of mice. (**C**) Daily body weight change of mice. (**D**) DAI score of mice over the entire experimental period. (**E**,**F**) Represent liver index and spleen index of mice, respectively. Data are mean ± SD of eight independent samples. ** *p* < 0.01; *** *p* < 0.001; **** *p* < 0.0001.

This study aimed to investigate the ameliorative effect of inactivated AKK PROBIO on DSS-induced mouse UC, with the experimental procedure detailed in Figure 1A. Consistent with the DSS group, the AKK_PRO group exhibited a gradual decline in food intake and body weight during the first 10 days of the experiment. Notably, while the DSS group continued to show persistent reductions in food intake and body weight from days 10 to 14, the AKK_PRO group displayed a marked recovery trend (*p* < 0.01, Figure 1B,C). The Disease Activity Index (DAI) score, a direct indicator of colonic injury severity, was significantly lower in the AKK_PRO group compared to the DSS group (*p* < 0.0001, Figure 1D). Additionally, inactivation of AKK PROBIO led to a downward trend in immune organ indices. Collectively, these findings suggest that inactivated AKK PROBIO effectively mitigates DSS-induced mouse UC.

### 3.2. Ingestion of Inactivated AKK PROBIO Alleviates Colonic Damage in DSS-Induced Mouse UC

**Figure 2:**
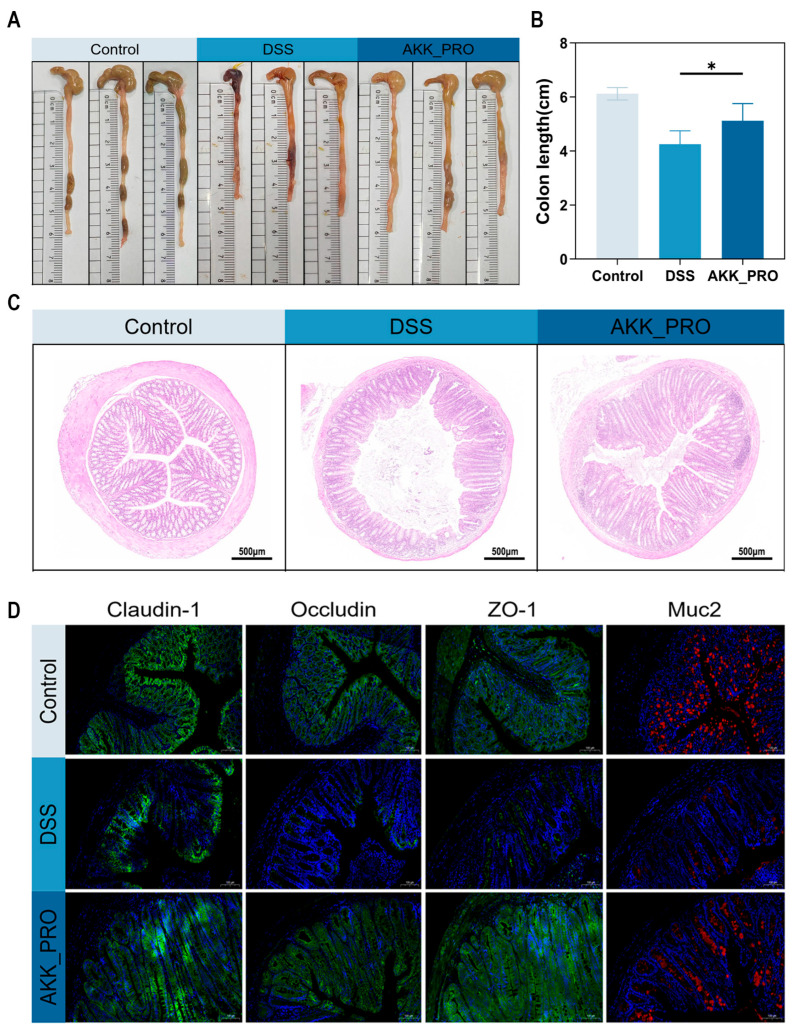
Inactivated AKK PROBIO mitigates pathological colon damage from DSS in mice. (**A**,**B**) Colon length of mice (*n* = 8 per group). (**C**) Representative images of H&E staining (*n* = 3 per group), scale bar: 500 μm. (**D**) Representative immunofluorescence images of colonic Claudin-1, Occludin, ZO-1 and Muc2 expression (*n* = 3 per group), scale bar: 100 μm. Data were shown as means ± SD. * *p* < 0.05.

The intestinal barrier plays a crucial role in maintaining gut homeostasis in mice. The experimental results showed that DSS treatment significantly reduced colon length in mice, whereas administration of inactivated AKK PROBIO markedly improved colon length (*p* < 0.05, Figure 2A,B). H&E staining revealed that the colonic tissue in the control group exhibited intact structure with well-arranged epithelial cells. In contrast, the DSS group displayed obvious pathological changes, including disorganized and disrupted epithelial cells, inflammatory cell infiltration, and damage to crypts and goblet cells. Notably, the AKK_PRO group showed significantly alleviated histopathological damage, with relatively preserved colonic architecture (Figure 2C). Immunofluorescence staining further assessed the expression of tight junction proteins (Claudin-1, Occludin, ZO-1) and mucin 2 (Muc2) in colonic tissues. Compared with the DSS group, AKK_PRO treatment markedly enhanced the fluorescence intensity of these proteins (Figure 2D). These findings demonstrate that inactivated AKK PROBIO effectively mitigates DSS-induced colonic injury and improves intestinal barrier function in mice.

### 3.3. Ingestion of Inactivated AKK PROBIO Reduces Inflammatory Cytokines and Alters Gut Microbiota Composition in DSS-Induced UC in Mice

**Figure 3:**
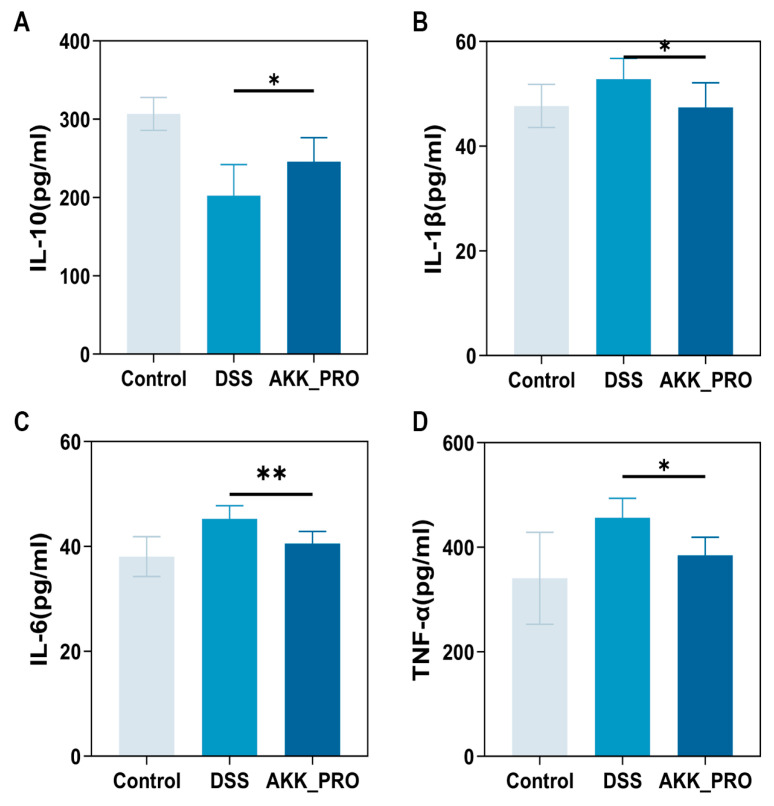
Inactivated AKK PROBIO corrects the inflammatory cytokine imbalance in DSS−treated mice. (**A**) The changes in IL−10. (**B**) The changes in IL−1β. (**C**) The changes in IL−6. (**D**) The changes in TNF−α (*n* = 8 per group). Data were shown as means ± SD. * *p* < 0.05, ** *p* < 0.01.

**Figure 4:**
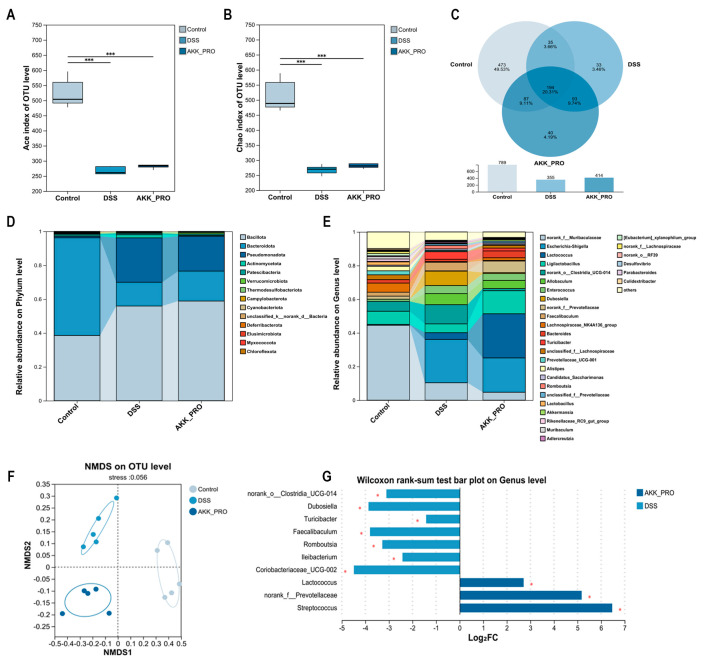
Inactivated AKK PROBIO counteracts DSS-induced gut microbiota dysbiosis in mice. (**A**,**B**) The Ace and Chao indices, respectively. (**C**) Venn diagram. (**D**,**E**) The microbial composition at the phylum and genus levels, respectively. (**F**) On−metric multidimensional scaling (NMDS) analysis. (**G**) Visualization and comparison of differential species between two groups. *n* = 5 per group. Data were shown as means ± SD. * *p* < 0.05, *** *p < *0.0001 .

The exacerbation of UC is typically associated with an imbalance in the inflammatory cytokine network and dysbiosis of the gut microbiota [23]. Analysis of inflammatory cytokines revealed that, compared to the DSS model group, the inactivated AKK PROBIO intervention group exhibited a significantly improved cytokine profile: the anti-inflammatory cytokine IL-10 was markedly elevated (*p* < 0.05, Figure 3A), whereas the pro-inflammatory cytokines IL-1β (*p* < 0.05, Figure 3B), IL-6 (*p* < 0.01, Figure 3C), and TNF-α (*p* < 0.05, Figure 3D) were all significantly reduced. 16S rRNA sequencing analysis of the gut microbiota in UC mice demonstrated that, compared to the healthy control group, both the DSS-induced group and the AKK_PRO intervention group exhibited a significant reduction in microbial α-diversity, as evidenced by a substantial decrease in both Ace and Chao indices (*p* < 0.001, Figure 4A,B). Further Venn diagram analysis showed that the control group harbored a total of 789 microbial species, significantly more than the 355 species in the DSS group and 414 species in the AKK_PRO group. Regarding group-specific microbial composition, the control group exhibited a differential microbial proportion of 49.53%, whereas the DSS and AKK_PRO groups accounted for 3.46% and 4.19%, respectively (Figure 4C). These findings collectively confirm that both DSS induction and AKK_BIO intervention significantly alter the overall composition of the gut microbiota.

At a more refined taxonomic-level, phylum-level analysis revealed that the gut microbiota of the control group was predominantly composed of *Bacillota*, *Bacteroidota*, *Pseudomonadota*, *Actinomycetota*, *Patescibacteria*, and *Verrucomicrobiota*. DSS treatment led to a significant increase in the abundance of *Bacillota*, *Pseudomonadota*, and *Actinomycetota*, accompanied by a marked reduction in *Bacteroidota*. Notably, intervention with inactivated AKK PROBIO reversed this trend, resulting in elevated abundances of *Bacillota*, *Bacteroidota*, and *Verrucomicrobiota*, alongside decreased levels of *Pseudomonadota* and *Actinomycetota* (Figure 4D). Genus-level analysis further demonstrated that, compared to the control group, the DSS group exhibited significant reductions in several beneficial bacterial genera, including *norank_f__Muribaculaceae*, *Ligilactobacillus*, *norank_f__Prevotellaceae*, and *Lachnospiraceae*_NK4A136_group, while pathogenic *Escherichia-Shigella* was markedly enriched. In contrast, intervention with inactivated AKK PROBIO exerted a selective regulatory effect, significantly decreasing the abundance of pathogen-associated genera (e.g., *norank_f__Muribaculaceae* and *Escherichia-Shigella*) while concurrently enhancing the relative abundances of beneficial genera, including *Ligilactobacillus*, *norank_f__Prevotellaceae*, *Lachnospiraceae*_NK4A136_group, *Bacteroides*, and *Akkermansia* (Figure 4E). The integrated analysis presented in Figure 4F further validated these differences in microbial composition, while the visualization of differentially abundant species between the DSS and AKK_PRO groups in Figure 4G exhibited strong consistency with the genus-level findings. Collectively, these results underscore the specific regulatory effects of inactivated AKK PROBIO on UC-associated dysbiosis.

### 3.4. Functional Remodeling of Gut Microbial Metabolism Through Inactivated AKK PROBIO Administration

**Figure 5:**
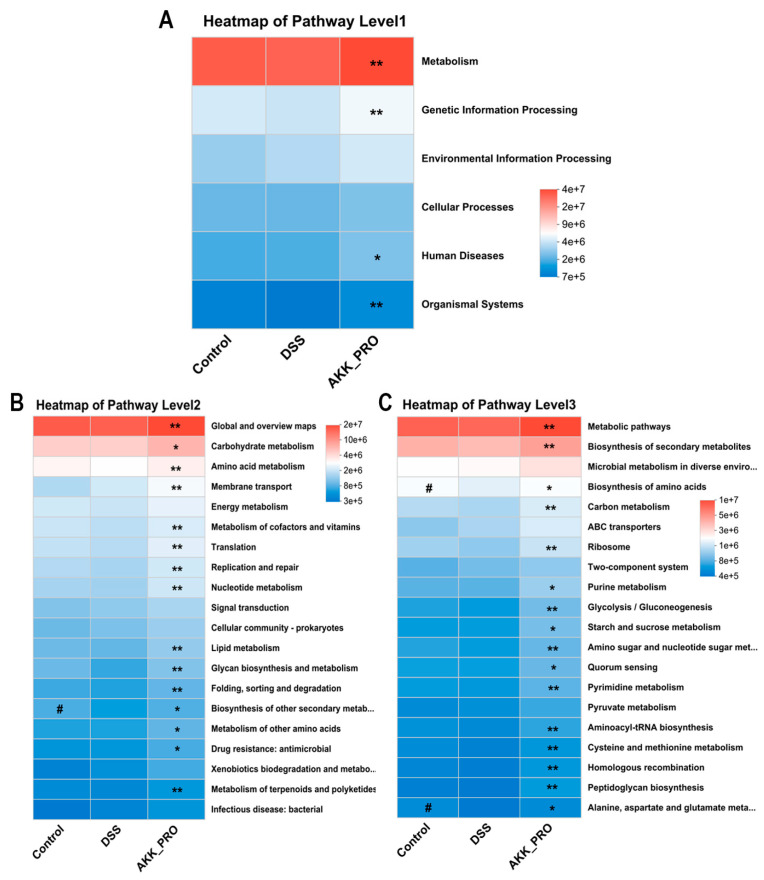
Functional remodeling of gut microbial metabolism through inactivated AKK PROBIO administration. (**A**) Heatmap of pathway level1. (**B**) Heatmap of pathway level2. (**C**) Heatmap of pathway level3. *n* = 5 per group. Data were shown as means ± SD. * indicates DSS vs. AKK_PRO group, # indicates Control vs. DSS group, * *p* < 0.05, ** *p* < 0.01, # *p* < 0.05.

Based on 16S rRNA gene amplicon sequencing data, we conducted a comprehensive functional profiling of gut microbiota using PICRUSt2 for KEGG pathway analysis. The heatmap visualization revealed substantial variations in metabolic pathway distributions across three hierarchical levels between DSS and AKK_PRO groups. At the primary classification level (Level 1), the AKK_PRO group demonstrated superior metabolic capacity, exhibiting significantly elevated relative abundances in both Metabolism and Genetic Information Processing pathways compared to the DSS group (Figure 5A). Delving into secondary pathways (Level 2), the AKK_PRO intervention showed pronounced enrichment in fundamental metabolic networks including Global and Overview Maps, Carbohydrate Metabolism, Amino Acid Metabolism, and Membrane Transport systems (Figure 5B). The tertiary pathway analysis (Level 3) further confirmed enhanced functional engagement of AKK_PRO-treated microbiota in pivotal biosynthetic processes such as Metabolic pathways, Biosynthesis of secondary metabolites, and amino acid biosynthesis pathways (Figure 5C). These observations collectively indicate that AKK_PRO administration potentially orchestrates a global activation of microbial metabolic circuitry, with particular emphasis on amplifying core biosynthetic machinery and energy transduction pathways, thereby modulating gut microbiota functionality through metabolic reprogramming.

### 3.5. Intake of Inactivated AKK PROBIO Increased SCFA Levels in DSS-Induced Ulcerative Colitis Mice

**Figure 6:**
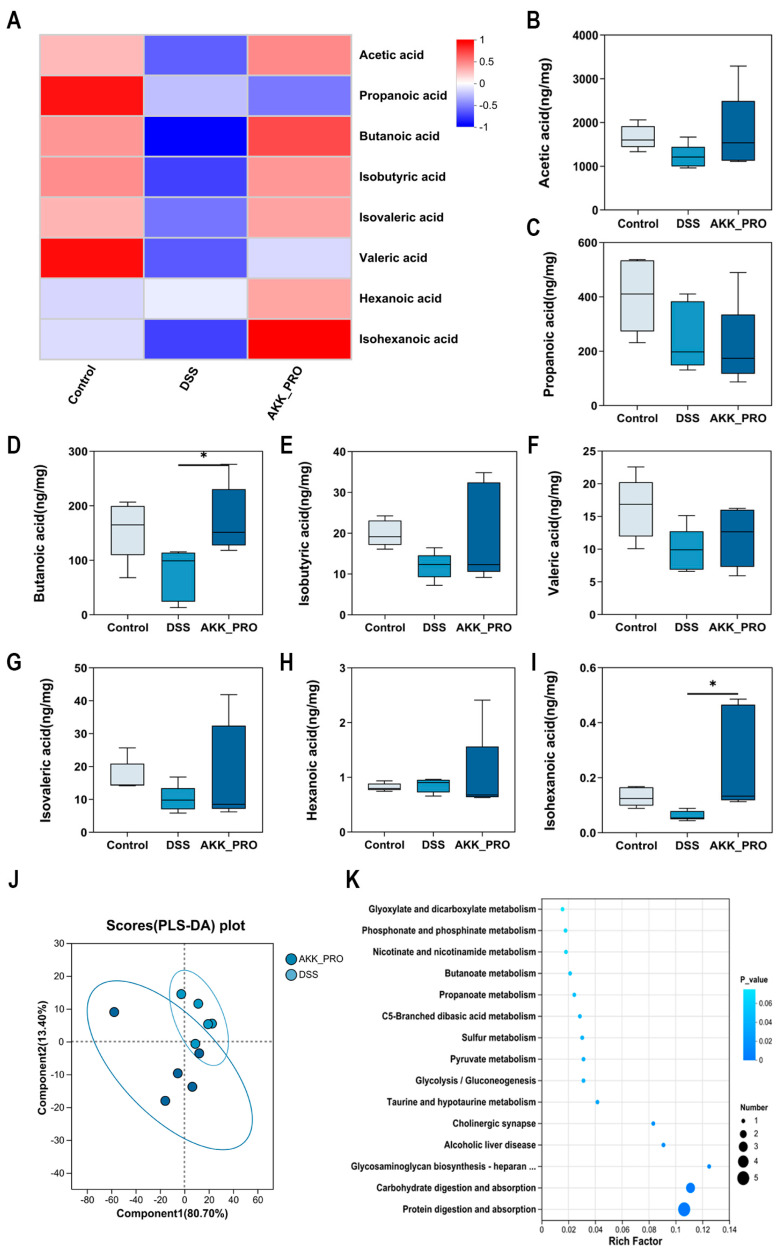
Inactivated AKK PROBIO boosted SCFA levels in a mouse model of DSS-induced colitis. (**A**) Overall metabolome clustering heat map. (**B**–**I**) Quantitative analysis of acetate, propionate, butyrate, isobutyrate, valerate, isovalerate, caproate, and isocaproate was performed. (**J**) Partial least squares-discriminant analysis (PLS-DA). (**K**) KEGG pathway enrichment analysis between DSS and AKK_PRO groups. *n* = 5 per group. Data were shown as means ± SD. * *p* < 0.05.

We employed gas chromatography–mass spectrometry (GC−MS) to quantitatively analyze SCFAs in murine cecal contents. Hierarchical clustering heatmap analysis revealed a marked reduction in total SCFA levels in the DSS model group compared to controls (Figure 6A). Intriguingly, administration of inactivated AKK PROBIO demonstrated a pronounced restorative effect on SCFA profiles. As illustrated in Figure 6B−I, quantitative assessment showed significantly elevated concentrations of butyrate and isocaproate in the AKK_PRO group relative to DSS-treated mice (*p* < 0.05). While acetate, isobutyrate, and valerate exhibited increasing trends post-intervention, these changes lacked statistical significance. Partial least squares−discriminant analysis (PLS−DA) demonstrated 80.70% separation along Component 1 between the DSS and AKK_PRO groups (Figure 6J), indicating substantial compositional divergence in their SCFA signatures. Subsequent KEGG pathway enrichment analysis uncovered multiple differentially regulated metabolic pathways between the groups (Figure 6K), providing mechanistic insights into the observed SCFA alterations.

### 3.6. Interrelationship Analysis Between Gut Microbiota, SCFAs, and Inflammatory Factors

**Figure 7:**
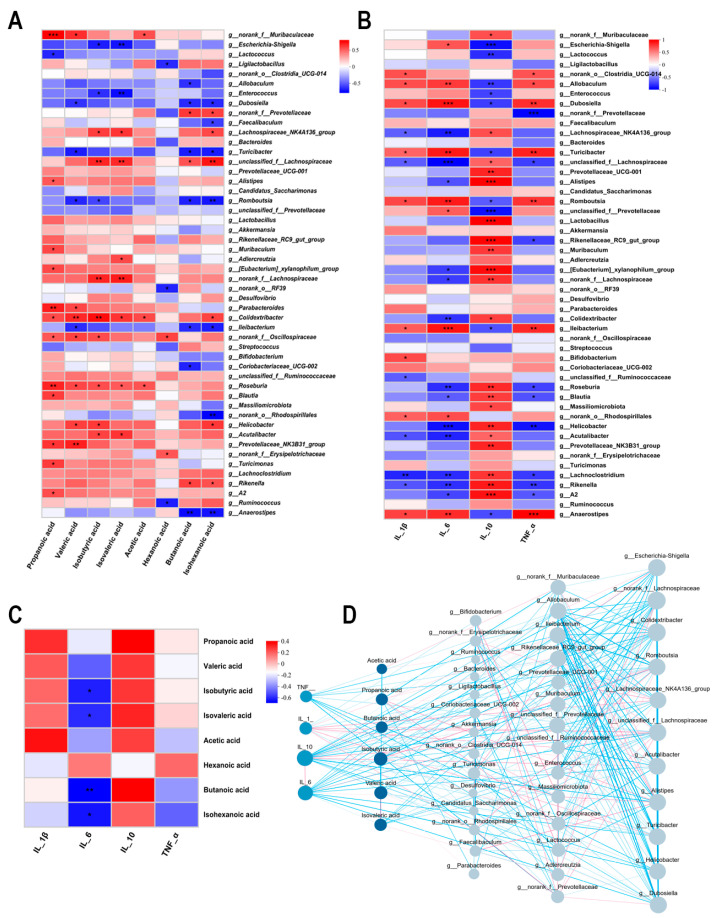
Interrelationship analysis between gut microbiota, SCFAs, and inflammatory factors. (**A**) The heatmap depicts the correlations between SCFAs and the gut microbiome. (**B**) The heatmap illustrates the associations between inflammatory cytokines and the gut microbiota. (**C**) The heatmap visualizes the relationships between inflammatory mediators and SCFA levels. (**D**) Network analysis of the correlations among SCFAs, gut microbiome, and inflammatory factors. *n* = 5 per group. Data were shown as means ± SD. * *p* < 0.05, ** *p* < 0.01, *** *p* < 0.001.

We systematically evaluated the tripartite interactions among gut microbiota, SCFAs, and inflammatory cytokines using Spearman correlation analysis. As illustrated in Figure 7A,B, both SCFAs and the anti-inflammatory cytokine IL−10 exhibited significant positive correlations with several beneficial bacterial genera (including *Lachnospiraceae*_NK4A136_group, *Blautia*, *Muribaculum*, and *Lactobacillus*), while demonstrating inverse associations with putative pathobionts (such as *Escherichia*−*Shigella* and *Enterococcus*). Intriguingly, pro-inflammatory mediators (IL−1β, IL−6, and TNF−α) displayed diametrically opposed correlation patterns with microbial taxa. Subsequent analysis revealed that SCFA concentrations positively correlated with IL−10 levels but were negatively associated with pro-inflammatory cytokines (IL−6 and TNF−α) (Figure 7C), a finding further corroborated by the results presented in Figure 7D. These consistent observations suggest that specific gut microbial communities may modulate host inflammatory responses through SCFA-mediated mechanisms, thereby providing novel insights into the gut microbiota–metabolite–immune axis in colitis pathogenesis.

## 4. Discussion

This study employed DSS to establish an experimental colitis model, wherein the compound induces intestinal barrier dysfunction by disrupting the mucosal layer and epithelial tight junctions. It is noteworthy that the pathological features elicited by DSS closely mirror those observed in clinical ulcerative colitis, primarily affecting the distal colon and rectum. These manifestations include the classic triad of weight loss, diarrhea, and hematochezia, accompanied by characteristic mucosal inflammatory infiltration and loss of barrier integrity [24]. Experimental data revealed that the inactivated AKK PROBIO intervention group exhibited significant therapeutic effects compared to the DSS group: food intake and body weight of the experimental animals improved, colon length increased by approximately 20%, the DAI decreased by about 62%, and immune organ parameters such as the splenic index tended to normalize. Histopathological evaluation further confirmed that the model group displayed extensive epithelial structural damage, crypt abscess formation, and goblet cell depletion—all hallmark pathological changes—whereas the AKK_PRO group maintained relatively intact mucosal architecture and preserved colonic tissue integrity. These findings systematically demonstrate, from macroscopic to microscopic levels, the multi-faceted ameliorative effects of inactivated AKK PROBIO on experimental colitis.

Numerous studies indicate that a hallmark pathological feature of UC is persistent mucosal inflammation in the colon. Consequently, preserving the integrity of the colonic mucus layer and suppressing excessive inflammatory responses have emerged as critical strategies for alleviating disease symptoms [12]. The colonic mucus layer can be subdivided into a dense inner stratum firmly adherent to the epithelial surface and a looser outer layer, with MUC2 serving as its core structural protein [25]. Under inflammatory conditions, patients often exhibit a reduced mucus layer thickness, which facilitates increased contact between gut microbes and their metabolites with epithelial cells. This proximity triggers immune activation and promotes the release of pro-inflammatory cytokines, thereby exacerbating the inflammatory cascade [26]. The present study demonstrates that intervention with inactivated AKK PROBIO significantly upregulates MUC2 protein expression in the colonic mucosa. Concurrently, this treatment markedly enhances the expression of the anti-inflammatory cytokine IL-10 while effectively suppressing the production of pro-inflammatory mediators such as IL-1β, IL-6, and TNF-α. These results suggest a potential involvement of the TLR4/NF-κB/NLRP3 axis, which warrants further mechanistic investigation [27]. Toll-like receptor 4 (TLR4), a key pattern recognition receptor in the innate immune system, can be activated by infiltrating pathogen-associated molecular patterns—such as lipopolysaccharide (LPS)—upon intestinal barrier compromise. This activation initiates downstream signal transduction, promoting the expression of inflammatory cytokines including IL-1β, IL-6, and TNF-α. NF-κB, a central transcription factor in TLR4 signaling, further binds to promoter regions of pro-inflammatory genes to amplify their transcription and intensify inflammatory responses [28]. Additionally, activation of the NLRP3 inflammasome catalyzes the maturation and release of IL-1β, establishing a positive feedback loop that perpetuates inflammatory escalation [29]. Based on these findings, we hypothesize that inactivated AKK PROBIO may attenuate the initiation and progression of colitis by modulating the TLR4/NF-κB/NLRP3 signaling axis.

In UC, aberrant mucin expression and concomitant inflammatory responses can lead to dysregulated tight junction (TJ) protein expression and increased intestinal permeability, thereby compromising gut barrier function [30,31]. Previous research has indicated that postbiotics play a crucial role in maintaining intestinal integrity through multiple mechanisms: firstly, by directly acting on intestinal epithelial cells to upregulate the expression of TJ proteins such as ZO-1, Occludin, and Claudin-1; secondly, by leveraging their antioxidant and anti-inflammatory properties to mitigate oxidative stress and inflammatory responses, thus reducing damage to the mucosal barrier; and thirdly, by competitively binding to intestinal surface receptors or secreting antimicrobial substances to inhibit pathogen colonization and growth, thereby indirectly strengthening barrier defense [1]. Thermally inactivated Streptococcus thermophilus significantly enhanced the structural integrity and functional recovery of the epithelial barrier by upregulating the expression levels of Occludin and ZO-1 [32]. Furthermore, postbiotics derived from Lactobacillus fermentum HF06 markedly strengthened the intestinal mucosal barrier [33]. In the present study, TJ protein expression was significantly reduced in the DSS group, whereas intervention with inactivated AKK PROBIO effectively reversed this downward trend.

Both clinical and preclinical studies have indicated that the amelioration of colonic symptoms, accompanied by intestinal barrier restoration and reduced inflammation, is often closely associated with the recovery of a healthier gut microbiota [30,34]. Given the potential role of postbiotics in promoting colonic homeostasis and modulating microbial communities, this study further analyzed the impact of inactivated AKK PROBIO intervention on the structure of the gut microbiota. The Ace and Chao indices are used to evaluate the species richness of the community [35]. However, no significant differences were observed in α-diversity indices between the DSS group and the AKK_PRO group. Venn diagram analysis revealed that although some differential taxa were present in both the DSS and AKK_PRO groups compared to the control group, their relative abundances were low (3.46% and 4.19%, respectively), suggesting that the improvement in colitis symptoms may not stem from drastic alterations in the gut microbiota [30]. Accordingly, we conducted an in-depth analysis of microbial composition at both the phylum and genus levels. The DSS group exhibited a marked increase in the abundance of Escherichia-Shigella and Enterococcus, both of which can exacerbate inflammatory responses by activating TLR pathways and promoting the production of pro-inflammatory cytokines [30]. Following intervention with inactivated AKK PROBIO, the abundance of these genera decreased, while beneficial genera such as *Ligilactobacillus*, *Lachnospiraceae*_NK4A136_group, *unclassified_f__Lachnospiraceae*, and *Akkermansia* showed increased expression. Previous studies have reported that *Ligilactobacillus* can directly inhibit the growth of potential pathogens through the secretion of metabolites like bacteriocins and lactic acid [36]; *Lachnospiraceae*_NK4A136_group and *unclassified_f__Lachnospiraceae* are capable of producing butyrate, acetate, and propionate, which supply energy to colonic epithelial cells and directly contribute to the repair and maintenance of the intestinal barrier [37]; meanwhile, *Akkermansia* enhances physical barrier function by promoting the formation of a thicker and more abundant mucus layer in the host and upregulating the expression of tight junction proteins such as Occludin [38]. Under conditions of gut dysbiosis, certain pathogenic bacteria or dietary components can stimulate the excessive release of TJ. Under normal physiological conditions, occludin polymers form a crucial sealing structure within the tight junction strands, working in concert with other proteins to create a selective barrier. However, the activation of TJ disrupts this synergistic interaction, causing occludin to dissociate from the junctional complex, thereby increasing paracellular permeability and consequently impairing barrier function [39].

Through PICRUSt2-based functional prediction analysis, we identified several KEGG pathways potentially involved in alleviating ulcerative colitis (UC). From the overall pathway architecture at Level 1 to Level 3, intervention with inactivated AKK PROBIO primarily enhanced functions related to metabolism. At Level 3, significantly enriched pathways included Metabolic pathways, Biosynthesis of secondary metabolites, and Biosynthesis of amino acids. The activation of these pathways may exert protective effects through multiple mechanisms: beneficial bacteria can directly inhibit or eliminate pathogenic species by synthesizing secondary metabolites such as bacteriocins, thereby ameliorating microbial dysbiosis; they are also capable of supplying essential trace elements to the host, promoting overall health and metabolic homeostasis. Furthermore, bacteria-derived amino acids may serve as an additional source for the host, supporting intestinal epithelial repair and regeneration processes [3,34,40].

A symbiotic relationship exists between the gut microbiota and SCFAs. As key end-products of microbial metabolism, SCFAs provide sustained nutritional support to both the microbial community and the host, collectively contributing to intestinal homeostasis. In this study, GC-MS-based metabolomic analysis of fecal samples revealed distinct differences between the DSS and AKK_PRO groups: the latter exhibited elevated levels of acetate, butyrate, isobutyrate, and valerate, with a particularly significant increase in butyrate concentration. Numerous studies have demonstrated that SCFAs can activate receptors such as GPR41 and GPR43, initiating downstream signaling cascades that modulate critical pathways including extracellular signal-regulated kinases, mitogen-activated protein kinases, and NF-κB [41]. Butyrate, in particular, promotes the differentiation of regulatory T cells (Tregs)—either through direct action on naïve CD4^+^ T cells or via indirect modulation of dendritic cell function—thereby enhancing immune tolerance [42]. Correlation heatmap analysis between gut microbiota and SCFAs indicated a strong positive association between SCFA production and genera such as *Bacteroides* and *Roseburia* [43]. Furthermore, SCFAs serve as a vital energy source for goblet cells, stimulating the synthesis of MUC2 protein and thereby supporting the formation of a more robust intestinal mucosal barrier [44]. Consequently, modulating the gut microbiota to enhance SCFA levels has emerged as a significant therapeutic strategy in the management of UC.

## 5. Conclusions

**Figure 8:**
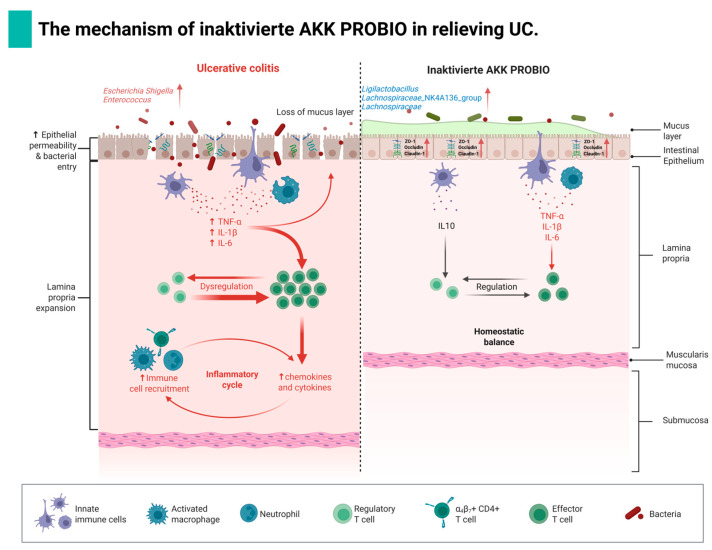
The overall mechanism of inactivating AKK PROBIO to alleviate UC (the graphic was created in Hongyan Zhang (2025) BioRender.com).

This study systematically investigated the therapeutic effects of inactivated AKK PROBIO on DSS-induced UC in mice. Our findings confirm our initial hypothesis that inactivated AKK PROBIO ameliorates UC pathology through synergistic multi-pathway mechanisms: firstly, it significantly enhances intestinal barrier function by promoting the expression of mucin MUC2 and facilitating the assembly of tight junction proteins (ZO-1, Occludin, and Claudin-1), thereby reducing mucosal permeability; secondly, it effectively modulates the inflammatory cytokine network by suppressing the expression of pro-inflammatory factors (TNF-α, IL-1β, and IL-6) while elevating anti-inflammatory IL-10 levels, leading to the remodeling of the immune microenvironment; thirdly, it restructures the gut microbiota ecosystem by increasing the relative abundance of beneficial bacteria (such as *Ligilactobacillus* and *Lachnospiraceae*_NK4A136_group), reducing colonization by opportunistic pathogens, and promoting the production of beneficial metabolites including SCFAs. The specific mechanism of action by which inactivated AKK PROBIO alleviates UC in mice is shown in Figure 8. Our findings validate the initial hypothesis by demonstrating that inactivated AKK PROBIO effectively alleviates DSS-induced colitis through three key mechanisms: intestinal barrier repair, immune regulation, and microecological balance restoration. However, the molecular mechanistic elucidation remains incomplete, particularly concerning the specific involvement of key signaling pathways such as TLR4/NF-κB/NLRP3. Future investigations should employ advanced techniques including Western blot, immunofluorescence co-localization, and transcriptomics to precisely map the regulated signaling nodes and identify direct interactions between bacterial components and host receptors.

Collectively, this study establishes a solid theoretical foundation for developing inactivated AKK PROBIO as a safe, food-derived postbiotic agent for UC management.

## Figures and Tables

**Table 1:** Calculated DAI score.

Score	Weight Loss (%)	Food Intake Decrease (g)	Fecal Consistency and Occult Blood
0	0–5	0–0.5	normal feces
1	5–10	0.5–1	loose stool
2	10–15	1–1.5	watery diarrhea
3	15–20	1.5–2	diarrhea with blood
4	over 20	over 2	watery diarrhea, with a lot of blood

## Data Availability

The original contributions presented in the study are included in the article, further inquiries can be directed to the corresponding author.
